# Distinctive lung function trajectories from age 10 to 26 years in men and women and associated early life risk factors – a birth cohort study

**DOI:** 10.1186/s12931-019-1068-0

**Published:** 2019-05-22

**Authors:** Wilfried Karmaus, Nandini Mukherjee, Vimala Devi Janjanam, Su Chen, Hongmei Zhang, Graham Roberts, Ramesh J. Kurukulaaratchy, Hasan Arshad

**Affiliations:** 1 Division of Epidemiology, Biostatistics, and Environmental Health, School of Public Health, University of Memphis, Memphis, TN USA; 2 Department of Mathematical Sciences, The University of Memphis, Memphis, TN USA; 3 Paediatric Allergy and Respiratory Medicine, Faculty of Medicine, University of Southampton, Southampton, UK; 4 Clinical and Experimental Sciences, Faculty of Medicine, University of Southampton, Southampton, UK; 5 David Hide Asthma and Allergy Research Centre, Newport, Isle of Wight UK

## Abstract

**Abstract:**

Pre-bronchodilator lung function including forced vital capacity (FVC), forced expiratory flow in 1 second (FEV_1_), their ratio (FEV_1_/FVC), and forced expiratory flow 25–75% (FEF_25–75_) measured at age 10, 18, and 26 years in the Isle of Wight birth cohort was analyzed for developmental patterns (trajectories). Early life risk factors before the age of 10 years were assessed for the trajectories.

**Method:**

Members of the birth cohort (1989/90) were followed at age 1, 2, 4, 10, 18, and 26 years. Allergic sensitization and questionnaire data were collected. Spirometry tests were performed and evaluated according to the American Thoracic Society (ATS) criteria at 10, 18, and 26 years. To identify developmental trajectories for FVC, FEV_1_, FEV_1_/FVC, and FEF_25–75_ from 10 to 26 years, a finite mixture model was applied to the longitudinal lung function data, separately for males and females. Associations of early life factors with the respective lung function trajectories were assessed using log-linear and logistic regression analyses.

**Results:**

Both high and low lung function trajectories were observed in men and women. FVC continued to grow beyond 18 years in men and women, whereas FEV_1_ peaked at age 18 years in female trajectories and in one male trajectory. For the FEV_1_/FVC ratios and FEF_25–75_ most trajectories appeared highest at age 18 and declined thereafter. However, the low FEV_1_/FVC trajectory in both sexes showed an early decline at 10 years. Lower birth weight was linked with lower lung function trajectories in males and females. Eczema in the first year of life was a risk factor for later lung function deficits in females, whereas the occurrence of asthma at 4 years of age was a risk factor for later lung function deficits in males. A positive skin prick test at age four was a risk for the low FEV_1_ trajectory in females and for the low FEV_1_/FVC trajectory in males.

**Conclusion:**

Men and women showed distinctive lung function trajectories and associated risk factors. Lower lung function trajectories can be explained by not achieving maximally attainable function at age 18 years and by a function decline from 18 to 26 years.

**Electronic supplementary material:**

The online version of this article (10.1186/s12931-019-1068-0) contains supplementary material, which is available to authorized users.

## Background

Lung growth is considered to end in women at about 18 and in men at about 20 years [1]. Accordingly, lung function peaks around these ages and declines thereafter. Following this, lung aging is associated with structural remodeling due to cell senescence resulting in reduced respiratory function [2]. During childhood and adolescence, it has been demonstrated that early lung function predicts later lung function and that distinctive developmental paths may exist [3–8]. In support of the developmental origins of health and diseases hypothesis [9], research has been shown that disadvantaged intrauterine condition, indicated by a reduced birthweight, are adversely associated with reduced lung function in children [10–18]. With the exception of one study [19], four studies reported an association between lower birth weight and reduced lung function [20–23]. Early life conditions, which may lower maximally attainable lung function, also include maternal smoking, childhood asthma, respiratory tract infections, and childhood smoke exposure [18, 24–26]. In addition, multiple studies have shown that reduced lung development early in life is a risk for lung diseases in later life [27, 28]. However, there is a need to test whether distinctive pattern of lung function development exist and then to determine factors predictive of different developmental patterns.

Analyzing patterns of lung function development over time will help us to detect unknown lung function trajectories with a higher risk for progression to COPD in adults. In the past, analyzing the development of lung function focused on arbitrary cut-off point of individual variability about the mean. For instance, patterns had determined subgroups by deviations from the mean lung function [8] or based on arbitrary taxonomic cutoffs using percentiles of growth, plateauing, and decline [29]. However, assessments based on such pre-defined deviations do not allow the identification of unknown, but yet meaningful trajectories [30]. In past studies, potential patterns were also defined a priori using a third variable, such as normal FEV_1_ in non-smokers compared to reduced FEV_1_ in smokers [8], or were identified using statistical methods designed to capture overall similarities independent of their development with age. Advanced statistical methods were rarely used to identify trajectories. Some statistical approaches established similarities with [25, 31] and without [32] taking the time order or age appropriately into account. Most recent studies on lung function identified latent classes [25, 32] but not trajectories addressing development over time. To our knowledge, only one publications modeled lung function trajectories allowing for non-linear developments of lung function in individuals [31] whose measurements followed a similar pattern over time [33, 34]. Some studies investigated trajectories of FEV_1_ [29, 31, 32], one explored the ratio of FEV_1_ and FVC [25], no study has yet examined trajectories in FVC and FEF_25–75_ and compared trajectories using these different markers. Although lung functions differ in men and women, past studies have not investigated both sexes separately. Appropriate knowledge about unknown sub-groups with different developments over time (trajectories) will support early detection of adverse developments and identification of risk factors important to prevent or mitigate them.

To gain better understanding of lung function trajectories, we analyzed pre-bronchodilator lung function of FEV_1_, FVC, FEV_1_/FVC, and FEF_25–75_ at ages 10, 18, and 26 years in the Isle of Wight (IoW) birth cohort. First, we explored developmental patterns by using trajectory analyses [33, 34], separately for male and female participants. Second, we focused on early life factors that could characterize different lung function trajectories at a later age. To consider an appropriate time order, only conditions ascertained before the first lung function measurement at 10 years of age were considered as risk factors. Hence, this work will not address factors such as adolescent smoking and pubertal changes that occurs parallel to the lung function observations (10–18 years). However, we will evaluate, whether the trajectories covering 10 to 26 years, are associated with diagnoses of asthma at 10, 18, and 26 years.

## Methods

### Study population

From January 1989 to February 1990 in the Isle of Wight (IOW), UK, the parents of every child (*n* = 1536) were approached to participate in a longitudinal study. After exclusion of adoptions, perinatal deaths and refusals, 94.8% (1456/1536) of all parents enrolled their newborn. The local research ethics committee approved the study. Informed written parental consent was obtained for all participants at recruitment and subsequently at each follow-up. The development of wheezing and asthma in the IOW birth cohort has been described in detail elsewhere [35–37]. Children were followed up at the ages of 1 (94.4%), 2 (84.5%), 4 (83.6%), 10 (94.3%), 18 years (90.2%), and as adults (26 years, 70.9%). The study represents a dynamic birth cohort; some children dropped out for one exam but re-joined the cohort at a later age.

### Questionnaires and clinical assessments

At birth, information was collected on maternal and paternal history of asthma, dog and cat ownership, and smoking during pregnancy. Maternal height and birth weight were transferred from the birth records. Cord serum was collected and immunoglobulin E (IgE) determined [38]. At the 1 and 2-year follow-up, information on duration of breastfeeding, introduction of formula and solids feeding, exposure to smoking and pets, and occurrence of chest infections was collected. At the 1, 2, and 4-year follow-ups, the medical investigator determined the presence of eczema and asthma based on symptoms over the last 12 months. For asthma the chronicity and frequency of wheeze over the last 12 months and physician-diagnosed asthma was used. For eczema, chronic or chronically relapsing, itchy dermatitis lasting more than 6 weeks with characteristic morphology and distribution were used [39], following Hanifin and Rajka criteria [40]. Height was measured at 4 years of life and every child was offered a skin prick test (SPT) with a standard battery of aero- (house dust mite (*Dermatophagoides pteronyssinus*), grass pollen mix, cat and dog epithelia, *Alternaria alternata*, *Cladosporium herbarum*) and food (milk, egg, soya, cod, wheat, peanut) allergens. Histamine (0.1%) in phosphate-buffered saline and physiologic saline were used, respectively, as positive and negative controls. The tests were read after 15 min, and a mean wheal diameter of at least 3 mm greater than the negative control was taken as positive. Any positive SPT was evaluated as indicator of an allergic sensitization. Information, on whether the child ever lived on a farm before age 10 years, was collected in the 10-year follow-up. [38]

To additionally inspect whether the prevalence of asthma at 10 and 18 years of age is related to different lung function trajectories (e.g., as a consequence of lower lung function), questions based on the International Study of Asthma and Allergies in Childhood (ISAAC) questionnaire [41] were completed by parents or study subject at age 10, 18, and 26 years. Asthma was defined as having “ever had asthma” and either “wheezing or whistling in the chest in the last 12 months” or “current treatment for asthma”.

### Pulmonary function tests

Pre-bronchodilator lung function tests were conducted at 10, 18, and 26 years of age. Forced vital capacity (FVC), forced expiratory volume in 1 second (FEV_1_), and forced expiratory flow when 25 and 75% of the FVC has been expired (FEF_25–75_) were measured using a Koko Spirometer and software with a portable desktop device (both PDS Instrumentation, Louisville, KY, USA). Spirometry was performed and evaluated according to the American Thoracic Society (ATS) criteria. The children or adults, respectively, were required to be free of respiratory infection for 2 weeks and not to be taking any oral steroids and were advised to abstain from any β-agonist medication for 6 h and from caffeine intake for at least 4 h [42].

### Trajectory analysis

To identify developmental trajectories for FVC, FEV_1_, the FEV_1_/FVC ratio, and FEF_25–75_ over time (10, 18, and 26 years), a finite mixture model was applied and implemented using PROC TRAJ macro in SAS® [43], separately for males and females. Trajectory parameters were estimated using the maximum likelihood approach built upon a binary logit model [44, 45]. PROC TRAJ is able to handle data that is missing completely at random [46], which fits the needs of our analyses, since we did not identify any factors explaining missingness at the three time points (10, 18, 26 years).

The objective of the trajectory analyses is to summarize distinctive features as parsimonious as possible [47]. To this end, parameter estimates for linear, quadratic, and cubic modeling terms for each subgroup were tested for each trajectory. We assumed at least two and at most four groups of lung trajectories, and each trajectory was inferred based on combinations of different order of polynomials. These steps were performed for males and females separately. To determine the number of trajectories, we followed the general recommendations of selecting the model with the smallest absolute Bayesian Information Criterion value that also minimizes overlap in the confidence intervals of adjacent trajectories while summarizing the distinctive features of the data in the most parsimonious model [44–46]. To further assess model adequacy, we ensured that the average of group-membership probabilities for individuals in each trajectory/group exceeded a threshold of 0.7 [46]. After selecting the best fitting model, individuals were assigned to one of the trajectories/groups based on their highest estimated group-membership probabilities. These categorical trajectory variables were then used in subsequent analyses.

### Statistical analysis of risk factors

All statistical analyses were performed using SAS statistical package, Version 9.2 (SAS Institute, Cary, NC, USA). For each observation period (age 1-or-2, 4, 10, and 18 years) differences in the occurrence of risk factors in the different trajectories, stratified for gender, were tested using Chi-square tests (two-sided *P* values). Applying log-linear and logistic regression models for two or three trajectories, respectively, we estimated the role of risk factors comparing low and high lung function trajectories for FVC and FEV_1_ and the odds of having lower lung function for three ranks of FEF_25–75_ trajectories. Risk ratios and odds ratios, respectively, and their 95% confidence intervals (95% CI) were estimated. All risk factors (maternal and paternal asthma, maternal smoking during pregnancy, cat and dog ownership, ever lived on a farm < 10 years, eczema in the first year, recurrent chest infections, skin prick test positivity, asthma at 4 years of age) were mutually adjusted for, when estimating their risks. We reduced the explanatory models by eliminating risk factors that did not have any effect if the removal did not change the risk/odds ratio of important factors by more than 10%. Eliminating some non-informative factors also increased the sample size, since the participation in some assessments (e.g., skin prick tests) was lower at some ages.

## Results

**Fig. 1:**
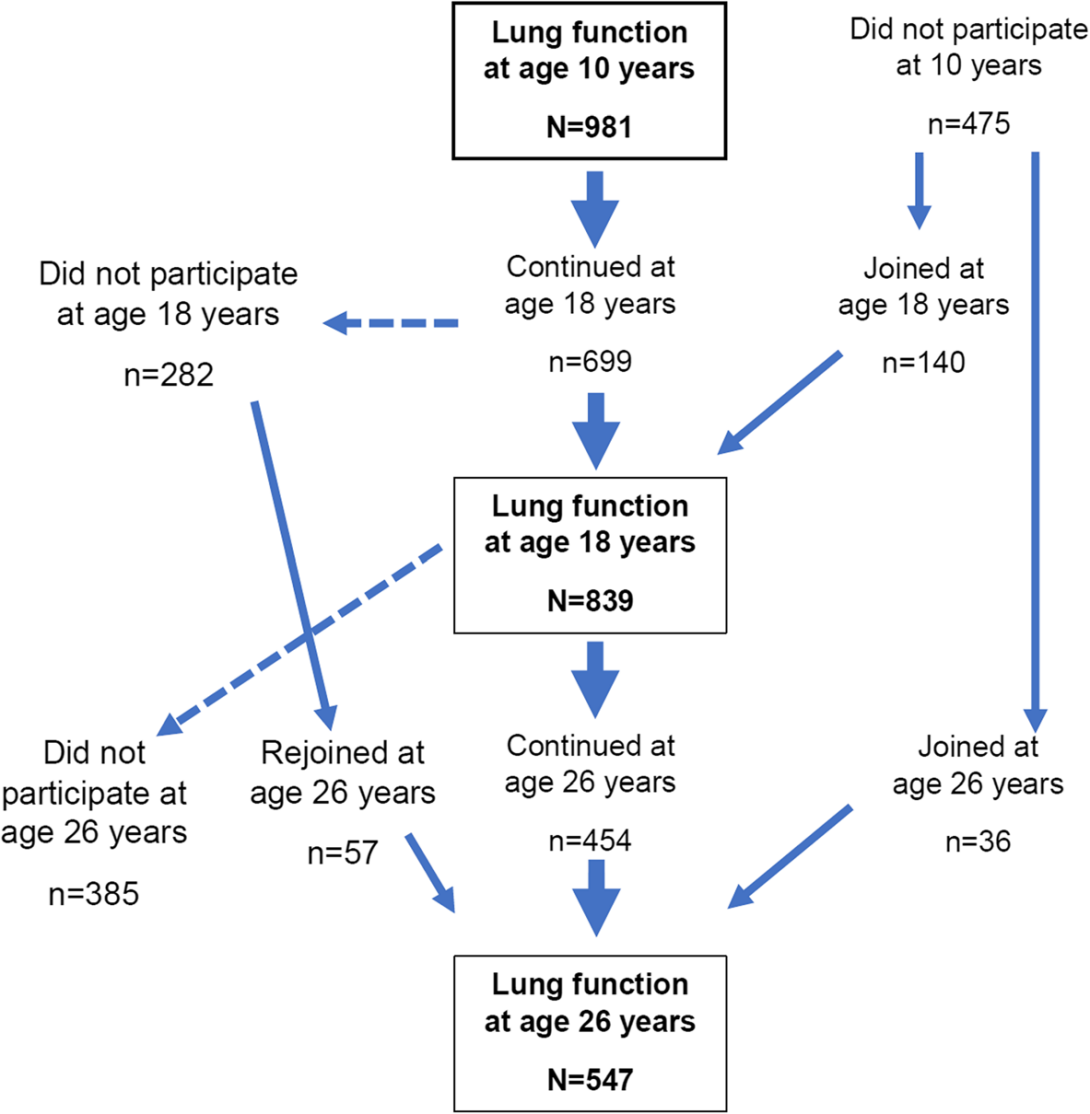
Flow chart of the participants in pulmonary function tests at age 10, 18, and 26 years in the Isle of Wight birth cohort

**Fig. 2:**
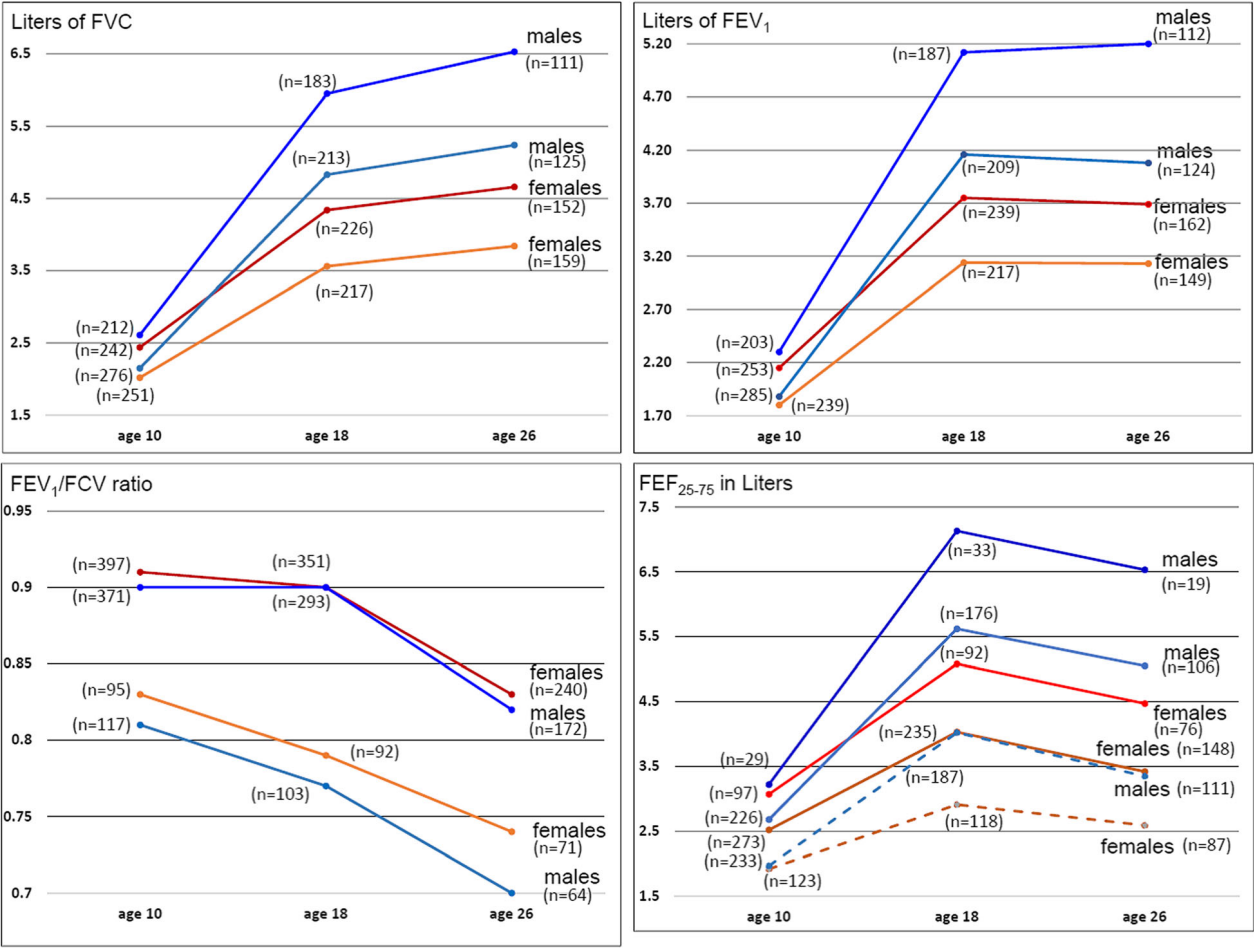
Lung function trajectories of Forced Vital Capacity (FVC), Forced Expiratory Volume in 1 second (FEV_1_), the ratio of the FEV_1_/FVC, and Forced Expiratory Flow 25–75% (FEF_25–75_) developments from age 10 to age 26 in female and male participants. Legend: The best fit for FVC and FEV1 trajectories in female and male participants were two quadratic patterns. For FEV_1_/FVC trajectories one linear and one quadratic trajectory gave the best fit, whereas for the three FEF_25–75_ trajectories in female and male participants modeling was most appropriate with three quadratic trajectories each

At total of 578 males and 580 females participated in at least one respiratory function test between 10 and 26 years (Fig. 1). Some did not participate at one test but joined again in a later assessment. Among male participants, 376 participants formed 2–3 trajectories for FVC, FEV_1_, FEV_1_/FVC, and FEF_25–75_. The respective number of female participants with trajectories was *n* = 432. The trajectories with the number of participants are shown in Fig. 2 and Additional file 1: Table S1. The sample sizes for the trajectories are presented in the column and the samples sizes for the different risk factors in the rows of Table 1.

**Table 1:** Factors related to trajectories (10–26 years) of forced vital capacity (FVC), forced expiratory flow in 1 second (FEV1), their ratio (FEV1/FVC), and forced expiratory flow (FEF25–75%) trajectories in male and female participants

Variables	Male participants								
	FVC trajectory		FEV_1_ trajectory		FEV_1_/FVC trajectory		FEF_25–75%_ trajectory		
	Low, *n* = 329%	High, *n* = 248%	Low, *n* = 329%	High, *n* = 248%	Low, *n* = 140%	High, *n* = 437%	Low, *n* = 273%	Medium, *n* = 267%	High, *n* = 37%
Maternal history of asthma (*n* = 573)	12.3	9.8	12.2	9.8	11.4	11.1	9.9	12.9	8.1
Paternal history of asthma (*n* = 569)	10.4	11.1	10.1	11.5	13.0	10.0	9.3	11.5	16.2
Maternal smoking during pregnancy (*n* = 574)	24.7	21.1	25.0	20.7	27.1	21.9	24.3	23.0	16.2
Owned cat at recruitment (*n* = 572)	31.4	36.1	31.4	36.1	35.7	32.6	34.3	32.2	35.1
Owned dog at recruitment (*n* = 572)	27.7	31.6	27.4	32.0	22.1*	**31.7**	**25.1**	34.5*	**24.3**
Ever lived on a farm < age 10 years (*n* = 511)	3.8	6.4	3.8	6.3	6.6	4.4	5.4	4.7	2.9
Eczema in the first year (*n* = 539)	11.0	11.7	11.9	10.6	14.8	10.2	12.5	10.2	11.4
Recurrent chest infections in years 1 or 2 (*n* = 492)	21.5	15.4	23.2*	**13.4**	23.4	17.6	21.9	16.0	20.0
Skin prick test positivity at age 4 (*n* = 429)	23.2	22.4	24.6	20.5	34.3*	**19.3**	**30.1**	14.6*	**32.1**
Prevalence of asthma at four 4 of age (510)	16.2	15.1	18.9*	**11.8**	25.4*	**12.6**	19.5*	**12.8**	**11.1**
Prevalence of asthma at 10 years of age (560)	20.1	15.3	20.9*	**14.2**	29.1*	**14.6**	22.4*	**15.3**	**5.7**
Prevalence of asthma at 18 years of age (523)	19.3	15.6	20.1	14.4	32.0*	**13.1**	25.2*	**11.6**	**5.7**
Prevalence of asthma at 26 years of age (*n* = 406)	15.3	12.5	17.8*	**9.4**	26.0*	**10.3**	20.2*	**9.0**	**6.7**
	mean or median ^γ^								
Birth weight (grams, *n* = 566)	3386*	**3539**	3373*	**3554**	3378	3476	3394*	**3475**	**3714**
Cord serum IgE (kU/L, *n* = 517)	0.06	0.06	0.06	0.06	0.06	0.06	0.06	0.06	0.22
Duration of breastfeeding (weeks, *n* = 524)	8*	**12**	8.5	12	12	11	12	10	13
Height at age 4 years (cm, *n* = 458)	102.8*	**106.0**	102.9*	**105.9**	104.6	104.1	103.6	104.5	105.6
Variables	Female participants								
	FVC trajectory		FEV_1_ trajectory		FEV_1_/FVC trajectory		FEF_25–75%_ trajectory		
	Low, *n* = 297%	High, *n* = 283%	Low, *n* = 278%	High, *n* = 302%	Low, *n* = 110%	High, *n* = 470%	Low, *n* = 141%	Medium, *n* = 323%	High, *n* = 116%
Maternal history of asthma (*n* = 577)	8.8	11.0	10.6	9.3	11.9	9.4	12.9	8.7	9.4
Paternal history of asthma (*n* = 574)	8.2	10.4	8.3	10.1	10.9	8.8	10.6	9.1	7.8
Maternal smoking during pregnancy (*n* = 579)	23.7	21.2	25.6	19.5	25.5	21.8	**26.2**	**23.9**	13.8*
Owned cat at recruitment (*n* = 579)	29.7	33.6	28.5	34.4	34.6	30.9	31.2	32.0	31.0
Owned dog at recruitment (*n* = 579)	28.0	29.0	29.6	27.4	31.8	27.7	32.6	27.6	25.9
Ever lived on a farm < age 10 years (*n* = 541)	3.7	7.5	4.3	6.7	9.6*	**4.6**	6.0	4.7	7.3
Eczema in the first year (*n* = 449)	12.5*	**5.6**	12.4*	**6.02**	12.5	8.3	**15.0**	**7.8**	5.4*
Recurrent chest infections in years 1 or 2 (*n* = 507)	17.9	15.1	17.2	16.0	17.0	16.5	17.2	17.7	12.8
Skin prick test positivity at age 4 (*n* = 488)	21.4*	**13.6**	22.2*	**13.3**	21.8	16.6	25.2*	**16.3**	**11.8**
Prevalence of asthma at four 4 of age (*n* = 510)	14.4	15.4	16.3	13.7	18.8	14.0	20.5*	**14.0**	**10.9**
Prevalence of asthma at 10 years of age (*n* = 561)	12.6	13.8	15.7	10.9	21.3*	**11.3**	21.7*	**11.2**	**8.2**
Prevalence of asthma at 18 years of age (*n* = 552)	19.6	20.6	24.9*	**15.7**	36.1*	**16.2**	35.7*	**16.4**	**10.9**
Prevalence of asthma at 26 years of age (*n* = 484)	16.4	17.5	20.2	14.1	**29.8**	**13.4**	28.6*	**15.6**	**5.9**
	mean or median ^γ^								
Birth weight (grams, *n* = 571),	3263*	**3453**	3270*	**3434**	3380	3350	**3269**	**3363**	3441*
Cord serum IgE (kU/L, *n* = 514)	0.06	0.06	0.06	0.06	0.06	0.06	0.6	0.6	0.6
Duration of breastfeeding (weeks, *n* = 542)	8	10	8	9	8	8	6.5	8.0	11.0
Height at age 4 years (cm, *n* = 462)	101.7*	**104.9**	101.7*	**104.8**	103.5	103.3	**102.1**	**103.5**	104.5*

* indicating statistical significance *p* ≤ 0.05 based on Chi-square tests for dichotomous variables and Wilcoxon (non-parametric) tests for continuous variables

^γ^ Median for cord serum IgE and duration of breastfeeding, mean for birth weight and height at age 4 years

Regarding number of participants, 578 male participants with at least one FVC measurement were statistically grouped in to two trajectories (*n* = 329 into the low and *n* = 248 into the high trajectory, Additional file 1: Table S2). Analogous analysis with 376 participants having at least two measurements also resulted in two trajectories. Upon comparing the membership resulting from these two analytical solutions, we found only two participants to switch their trajectories (Additional file 1: Table S2). Similarly, in female participants with at least one FVC measurement, 580 were included in two trajectories. With at least two measurements, 432 females were grouped into two trajectories. Only three of 432 female participants changed the trajectory (0.07%). A change of less than 1% is too small to expect different finding focusing on trajectories with at least two respiratory tests. However, a smaller proportion of participants in the trajectories with two required lung function tests would justify additional descriptions of the characteristics of these trajectories. Hence, we investigated the variables describing the characteristics of trajectories in Table 1 in the reduced samples, separate for male and female participants; however, only minor differences were found. Since there are no major modifications between these two solutions, we kept the original trajectory analyses, since the reduction in sample size is a larger trade-off. Regarding FEV1 and FEV1/FVC, the analyses of trajectories with at least one or at least two observations showed identical findings as described above (Additional file 1: Table S2). The analyses FEF25–75 revealed nearly identical results (data not shown).

Figure 2 shows that two (high/low) trajectories were identified for FEV_1_, FVC and FEV_1_/FVC ratio and three (high, medium and low) for FEF_25–75_. All trajectories of the four lung function markers display a distinct ranking after 18 years of age. Only for FEV_1_/FVC trajectories the ranking was already detectable after 10 years of age (Fig. 2). The growth period between 10 and 18 years seems to produce more differences: a high proportion of children did not reach a maximally attainable function at age 18 years. Except for the FEV_1_/FVC ratio, males improved their lung function between 10 and 18 years.

In addition to larger lung function in males, males and females have different trajectory pattern. In the development from 10 to 18 years, due to steeper increases, some trajectories of male participants cross those of females (Fig. 2). In general, FVC seems to grow from age 10 to 26 years, whereas FEV_1_ increases between 10 and 18 years and remains stable or slightly decreases after 18 years. Consequently, their ratio (FEV_1_/FVC) peaks at age 18 and decreases after this age in most trajectories. The low FEV_1_/FVC trajectory in both men and women (with about 20% of all participants) showed a steeper decline after the 10-year measurement, suggesting that individuals in this pattern show worsening airflow obstruction with increasing age. A similar pattern was seen for FEF_25–75_ in all six trajectories (Fig. 2) in males and females. The trajectories of FEF_25–75_ have a clear pattern of increase until 18 years and decline thereafter (Fig. 2). Interestingly, the number of subjects in the high FEF_25–75_ trajectory was the smallest (*n* = 19 to 33 in males, *n* = 92 to 76 in females, Fig. 2, Additional file 1: Table S1) among all FEF_25–75_ trajectories. The medium- and low-level trajectories constituted the largest groups.

The possibility of an accelerated decline was tested by comparing lung function at age 26 and age 18 (ΔFEV1/FVC = FEV1/FVC at age 26 - FEV1/FVC at age 18, ΔFEF25–75 = FEF25–75 at age 26 - FEF25–75 at age 18) for the two trajectories. A delta statistically significant different from zero comparing low with high trajectories would demonstrate a significantly stronger decline. Controlling for birth weight and height achieved at age 18 years, the analyses showed that in females the low FEV1/FVC trajectory showed a significantly stronger decline (*p* = 0.0001), but not in males. Similarly, for FEF25–75, the lower trajectory in females but not the middle trajectory demonstrated a steeper decline than the higher reference trajectory. No such effect was seen in males.

In male participants, the FVC trajectories and the FEV_1_ trajectories (two trajectories each, Fig. 2) showed a considerable overlap: 84.5% in males (79.8% in females) with a low FVC trajectory also had a low FEV_1_ trajectory (Table 2). In males, 79.4% had trajectories with both higher FVC and FEV_1_ values and 85.5% of females. Regarding agreements between the low trajectories of FEV_1_/FVC and three trajectories of FEF_25–75_ there is good agreement in both men and women. Of the medium and high FEF_25–75_ trajectories, only 7.4% had a low FEV_1_/FVC trajectory in women, and 2.3% in men.

**Table 2:** Agreement between lung function trajectories in male and female participants

	Male participants			Female participants		
	Low FVC-trajectory (*n* = 329) column percent	High trajectory (*n* = 248) column percent		Low FVC-trajectory (*n* = 297) column percent	High trajectory (*n* = 283) column percent	
Low FEV_1_ trajectory	84.5	20.6		79.8	14.5	
High FEV_1_ trajectory	15.5	79.4		20.2	85.5	
	Male participants			Female participants		
	Low FEF_25–75%_ trajectory (*n* = 139) column percent	Medium FEF_25–75%_ trajectory (*n* = 267) column percent	High FEF_25–75%_ trajectory (*n* = 37) column percent	Low FEF_25–75%_ trajectory (*n* = 141) column percent	Medium FEF_25–75%_ trajectory (*n* = 323) column percent	High FEF_25–75%_ trajectory (*n* = 116) column percent
Low FEV1/FVC trajectory	49.1	2.3	0	61.0	7.4	0
High FEV1/FVC trajectory	50.9	97.8	100.0	39.0	92.6	100.0

Inspecting crude associations, child’s birth weight and height at 4 years of age were significantly differently distributed mainly on FVC- and FEV_1_-trajectories in men and women (Table 1). Duration of breastfeeding, indicating a reduction in the risk ratio per additional week of feeding for being in the low FVC trajectory, played a role in males. The prevalence of eczema in the first year of life was higher in females with low FVC, FEV_1_, and FEF_25–75_ trajectories, whereas in males, recurrent chest infection was crudely associated with a lower FEV_1_-trajectory. Positive skin prick tests and asthma at 4 years of age were associated with various lower lung function trajectories in males and females (Table 1).

Considering various potential early life risks factors adjusting for others in log-linear (dichotomous outcomes: FVC, FEV_1_ and FEV_1_/FCV trajectories) and logistic regression analyses (three-level outcome of FEF_25–75_ trajectories), birth weight and positive skin prick tests at 4 years of age were the only factors that were linked to lower trajectories in males and females (Table 3). The higher the birth weight, the lower the risk/odds of having low trajectories; the only exception is FEV_1_/FVC trajectory in women. Having a positive allergic sensitization at 4 years of age was a risk for being in a low FEV_1_ trajectory in females and for a low FEV_1_/FVC trajectory in males. Interestingly, the occurrence of eczema in the first year of life (8.4% in females, 10.7% in males) was a risk factor for females, whereas the occurrence of asthma in males at 4 years of age (14.1% in females, 15.7% in males), was a risk factor in males for being in lower FEV_1_ and FEV_1_/FVC trajectories.

**Table 3:** Risk ratio of early life risk factors for developing pulmonary function test trajectories

Variables	Male participants				Female participants			
	Low FVC-trajectory (*n* = 661)	Low FEV_1_-trajectory (*n* = 501)	Low FEV_1_/FVC trajectory (*n* = 480)^a^	Low and medium FEF25–75% trajectories (*n* = 480)	Low FVC-trajectory (*n* = 654)	Low FEV_1_-trajectory (*n* = 468)^a^	Low FEV_1_/FVC trajectory (*n* = 733)	Low and medium FEF25–75% trajectories (*n* = 542)
	Risk Ratio (95% CI)	Risk Ratio (95% CI)	Risk Ratio (95% CI)	Odds Ratios (95%CI)	Risk Ratio (95% CI)	Risk Ratio (95% CI)	Risk Ratio (95% CI)	Odds Ratios (95%CI)
Birth weight (change in risks/odds per kilogram)	0.87 (0.78, 0.96)	0.87 (0.78, 0.97)	0.71 (0.52, 0.98)	Low vs. high: 0.27 (0.13, 0.55) medium vs. high 0.36 (0.18, 0.75)	0.78 (0.70, 0.86)	0.69 (0.57, 0.83)	–	Low vs. high: 0.48 (0.29, 0.8) medium vs. high 0.73 (0.47, 1.14)
Duration of breastfeeding (change in risks/odds per week)	0.995 (0.991, 0.999)	–	–	–			–	
Eczema in the first year of life	–	–	–	–	1.35 (1.12, 1.63)	1.39 (1.11, 1.75)	1.66 ¥ (1.001–2.77)	Low vs. high: 3.55 (1.36, 9.26) medium vs. high 1.6 (0.63, 4.03)
Skin prick test positivity at age 4	–	–	1.64 (1.14–2.36)	–		1.32 (1.07, 1.63)	–	
Asthma at four 4 of age	–	1.15 (1.01, 1.32)	1.68 (1.17, 2.43)	–			–	

^a^Lower number of participants, since Skin prick test at 4 years was involved

¥ No effect of eczema in the first year of life, when birthweight was in the model. However, eczema in the first year of life gained statistical significance, when height at 4 years of age was controlled for instead of birth weight

As others we also observe that most of the lung function trajectories we identified (e.g., FEV1 and FVC trajectories) in men and women are dependent on height at the respective age of measurement, as demonstrated in Additional file 1: Table S3 for the growth (height difference) between 10 and 18 years. However, growth and height are associated with multiple prenatal and early childhood exposures [48, 49]. Additionally, height effects vary with sex. In females, puberty stops the growth of the legs [50], which is partially compensated by trunk growth. This results in a lower sitting height to leg length ratio or higher trunk to leg ratio, respectively, in women than in men [50, 51]. Correspondingly, women with early menarche have a lower lung function [52]. Thus, for all four lung function markers, half of the associations seen for birth weight are eliminated once height is controlled for (Additional file 1: Table S3). Since height is correlated with birth weight, gender, and age at puberty, it is not appropriate to adjust lung function measurements for height when focusing on etiologic respiratory research of early life risk factors (unlike in a clinical setting). In etiologic respiratory research, we need to consider, that growth (height) is a consequence of early life factors and pubertal changes. Thus, adjusting lung function markers for height and sex will diminish the association of birthweight and other early life determinants with lung function and hinder their identification as potential risk factors. However, in a clinical setting, to determine abnormal values, we may need to correct for height, age and sex [53].

Asthma at age 10, 18, and 26 years and lung function were ascertained concomitantly, hence, asthma at age 10, 18, and 26 years cannot serve as risk factor. Nevertheless, to evaluate whether lung function trajectories are associated with asthma diagnoses at age 10, 18, and 26 years of age, we also inspected the prevalence of asthma in the various trajectories. Having a lower lung function trajectory, on average, was associated with a doubled prevalence of asthma at age 10 and 18 years in women and men (Table 1). The association was stronger for the trajectories of FEV_1_/FVC and FEF_25–75_. The prevalence of asthma nearly tripled for the three FEF_25–75_ groups.

## Discussion

Analyses of FVC, FEV_1_, FEV_1_/FVC ratio and FEF_25–75_ from 10 to 26 years, stratified for gender, revealed distinct developmental trajectories in the IoW birth cohort. FVC continues to grow throughout from 10 to 26, trajectories of FEV_1_ and FEF_25–75_ showed an increase between 10 and 18 and decline thereafter, while FEV_1_/FVC ratio trajectories declined from 10 to 26 in all except the high trajectory in male (which declined after 18 years of age). Some cohort members did not achieve maximally attainable lung function for FVC, FEV_1_, and FEF_25–75_; participants of other trajectories showed an accelerated decline. The trajectories based on FEV_1_/FVC differed from other trajectories in that these showed a consistent decline from age 10 to 26. This is largely explained by a proportionately higher gain between 10 and 18 years in FVC than FEV_1_ and relatively less decline between 18 and 26 years. As a composite of other measures, an optimal trajectory for FEV1/FVC ratio is difficult to rationalize. It can indicate a mismatch; for instance, if someone with low FEV_1_ has a higher FVC suggesting an excessive narrowing of the airway lumen (obstruction), which is captured by the FEV_1_/FVC ratio. In accordance with this explanation, our findings show agreement between FEV_1_/FVC and FEF_25–75_ as complementary signals of airflow obstruction (Table 2). Since the FEV_1_/FVC ratio may act as sensitive indicator of airway diameter, it provides a focused signal of airflow obstruction that may align with the propensity to develop COPD in later life.

Our trajectory analyses were based on participants with at least one pulmonary function test. When we investigated participants with two tests, there were only minimal changes comparing the two alternative trajectory solutions (Additional file 1: Table S2). Second, the characteristics shown in Table 1 varied little between these two solutions with different sizes of the analytical samples. Hence, it is justified to focus on the trajectory solution based on one measurement to attain maximum sample size.

Findings regarding when the effects of early life risk factors such as parental smoking manifest themselves, are in dispute. For instance, one study, focusing on FEV_1_/FVC tests at different ages showed that effects of parental smoking only become obvious at 26 years of age [54]. Against that, Bui at al. using trajectory analyses of lung function demonstrated that maternal smoking was a risk factor already for an ‘early below average lung function trajectory’ [31]. Thus, investigating early life risk factors in the context of lung function development from childhood to young adult life is critically important as a first step in preventing long term lung damage. Focusing on an appropriate time-order and restricting to risk factors before the first lung function measurement showed that the lung function in some participants may be primed from an early age for a higher risk of not reaching the optimal plateau and an early decline in lung function, possibly establishing a higher risk of COPD later in life [55]. Important early life factors were birth weight, duration of breastfeeding, eczema in the first year of life in females, and asthma at 4 years in males. In addition, in both genders, a positive skin prick test at age 4 years was linked to lower FEV_1_ and FEV_1_/FVC trajectories, respectively.

Of the five prior lung function studies that identified trajectories [8, 25, 29, 31, 32], three used modern statistical approaches to identify lung function patterns (Tucson Children’s Respiratory Study, the combined analyses of Manchester Asthma and Allergy Study (MAAS), the Avon Longitudinal Study of Parents and Children (ALSPAC), and the Perth Infant Asthma Follow-up (PIAF), and the Tasmanian Longitudinal Health Study [TAHS]). In these five cohorts ages range from 1 month to 18 years in PIAF, 5–6 years in MAAS, 8–24 years in ALSPAC, 5 to about 30 years in CAMP, [29] 11–32 years in the Tucson Children’s Respiratory Study, 6–53 years in TAHS, 21–40 years in the Framingham Offspring Cohort, 24–40 years in the Copenhagen City Heart Study, and 31–75 years in the Lovelace Smokers Cohort. Prior studies used one marker of lung function. Whereas the Tucson Children’s Respiratory Study focused on FEV_1_/FVC, all other investigations used FEV_1_. In younger children in the PIAF study, the Maximal Expiratory Flow adjusted for functional residual capacity levels (V’max/FRC) was additionally used to identify developmental patterns of lung function.

Different approaches have been applied to identify typical lung development trajectories. Clustering of the random effects for slopes and intercepts determined from linear mixed models adjusted for effects of sex were used for the Tucson birth cohort by Berry et al. [25]. The combined analyses of the MAAS, ALSPAC, and PIAF cohort data on FEV_1_ also employed random intercepts and coefficients from linear mixed models to examine trajectories with no or linear changes over time [32]. Adjusting for random intercept and sex in linear mixed models will reduce the variance of lung function measurements at different ages by extracting individually determined differences related to underlying pattern (intercept) and sex. Hence, using adjusted lung function data will reduce the chance to detect physiologically meaningful trajectories [56]. Thus, an unsupervised modeling strategy seems to be required. Such an unsupervised group-based trajectory modelling was used by the Tasmanian Longitudinal Health Study (TAHS) to model lung function from age 7 to age 53 years [31]. We also applied an unsupervised group-based trajectory method, since we believe that it is essential to administer unsupervised approaches to gain better understanding of lung function development separate in males and females, not to adjust for sex and not to exclude the effects of intercepts at different times of the lung function tests.

Regarding potential risk factors, maternal and paternal asthma and also maternal smoking during pregnancy as emphasized in other studies [57, 58], were not important for the pulmonary function trajectories in this cohort, nor were repeated chest infections as shown by others [58, 59]. Prior studies have documented that lower birth weight is associated with reduced lung function [20–23]. Accordingly, using birth weight in continuous scale, an increase in birth weight was inversely related (RR or OR < 1) with lower level trajectories of nearly all lung function markers (Table 2). It is possible, that the effects of parental asthma and maternal smoking are masked, when we controlled for birth weight, since the latter may act as intervening variable. However, of these three factors only maternal smoking affected offspring’s birth weight (maternal smoking: 3253 g, no smoking: 3449 g, *p* < 0.0001, Kruskal-Wallis Test) and thus may have an indirect effect on lung function, as has recently been shown in this cohort by another publication [18].

Although previously reported for the IoW birth cohort that offspring asthma was associated with a history of asthma in same sex parents, [60] we did not find any link of maternal or paternal history of asthma with lung function trajectories of their children. A lack of associations was also reported for the CAMP project, in which all children had asthma, [29] and in the TAHS study, in which parental asthma did not contribute to the risk profile [31]. The role of parental asthma was not addressed in the ALSPAC and MAAS study [32]. Only in the Tucson Children’s Respiratory Study, it was reported that maternal history resulted in an increased proportion (20%) of the low FEV_1_/FVC trajectory, compared to 9.9% in the normal trajectory group. No such associations were seen for asthma of the father. It remains to be evaluated in future studies, whether lung function trajectories can be explained by a history of parental asthma.

Breastfeeding seems to influence lung volume (FVC) in males, which may be related to an increased volume due to suckling by the child [42]. Early asthma was, in agreement with prior studies [61], associated with lower FEV_1_ and FEV_1_/FVC trajectories in males. Surprisingly, this association was not seen in females. Such a gender difference was reported before [62]. A novel finding of this stratified analysis is that, in women, eczema in infancy is a risk for lower FVC and FEV_1_ trajectories. It is possible that a shared unknown other risk factor for both eczema and lung function may establish such an association. Since we adjusted for a possible effect of a positive skin prick test, allergic sensitization is not a candidate of such a shared risk factor. However, polyunsaturated lipids early in life may present such a common risk factor [63]. Regarding the effect of allergic sensitization (skin prick test, SPT) it has been previously reported that sensitization is related to lower lung function [64]. In the IoW cohort, a positive SPT at age 4 is related to a lower FEV_1_ trajectory in females and a lower FEV_1_/FVC trajectory in males (Table 2).

As others have documented, early impairment of lung function is associated with asthma in later life [65, 66]. In our study, asthma at 4 years predicted lower lung function trajectory in males at 10–26 years of age. It is not possible to determine the cause and effect, as we do not have lung function measurement in early childhood and hence, an onset of lung function deficit may have been initiated after or before 4 years of age in those with asthma diagnosed at 4 years. It is likely that participants with low lung function trajectory had some lung function deficit in early childhood. For instance, the Tucson Children’s Respiratory Study analyzed the FEV_1_/FVC ratio in 599 participants at comparable ages (between 11 to 32 years) to the Isle of Wight study [25]. In this study, individuals with a persistently low trajectory demonstrated lower lung function during infancy and at age 6 years.

In the combined analyses of MAAS, ALSPAC, and PIAF, Belgrave et al. investigated trajectories of FEV_1_ and found 4 distinct trajectories; persistently high; normal; below average; and persistently low *N* = 2632 [32]. Similar to participants belonging to the low trajectory in our study, the persistently low trajectory subject failed to achieve the maximal attainable lung function by 18 years, beyond which a decline ensued. This is critically important as longitudinal studies have demonstrated that those who do not achieve a normal FEV_1_ in young adult life are at considerable high risk of COPD [8]. Further support for the early origins of COPD comes from the Tasmanian Longitudinal Health Study which showed the lower lung function trajectories of FEV_1_ contribute 75% to those diagnosed with COPD [31]. Allergic sensitization was a risk factor for low FEV_1_ trajectory in both these populations, as it was in our study in females. In males, we found that allergic sensitization constituted a risk for the low FEV_1_/FVC trajectory.

One strength of our study is the application of an unsupervised modeling strategy that did not require us to use arbitrary cutoffs to define deviant lung function. In addition, our models were flexible in that they allowed changes in lung function over time to vary between the different lung function trajectory classes. Using an appropriate time order in the occurrence of risk factors and repeated lung function data from a prospective birth cohort, allowed us to examine the relationships between early life factors and lung function trajectory through early adulthood. In addition, we use a wide base of four lung function markers and compared their trajectories and related early life risk factors. Attrition from 10 year (*n* = 981) to 18 years (*n* = 839) to 26 years (*n* = 547) imposes some caution generalizing the lung function findings. However, since there was no conditional pattern of missingness, all data were used to model the trajectories. The current investigation is limited to three lung function tests between 10 and 26 years of age; however, only the TAHS offered a wider age range with pulmonary function test between 7 and 53 years [31].

While prior studies investigated only one marker, without stratification by gender, this investigation provides trajectories for four different lung function markers (FVC, FEV_1_, FEV_1_/FVC, and FEF_25–75_) separately for male and female participants. Whereas the combined analysis of MAAS and ALSPAC data only allowed linear trajectory patterns [32] restricting the shape of the patterns, this investigation included mixtures of other developments (e.g., linear and quadratic) and found non-linear patterns that considerably explained the development of lung function over time (Fig. 2). Despite large volume and flow differences (on average 1 liter larger values in males than females), the overall pattern of lung function trajectories in male and female participants was similar (Fig. 2). In contrast to Tucson Children’s Respiratory study, which only focused on FEV_1_/FVC showing no gender differences [25], our findings suggest that we need to analyze male and female lung function separately. First, the proportion of trajectories varies by gender. For instance, 47.3% of males are in the low FEF_25–75_ trajectory, but only 24.3% of female participants (Table 1, *p* < 0.001). Second, some risk factors such as eczema in the first year of life and asthma at age 4 years vary by sex. In addition, our study focused on early life risk factors and did not address concurrent risks to avoid a possibility of reverse causation. Given this setting, we found a lower birth weight to be an important risk factor. However, only one other trajectory study also identified lower birth weight [32], others lacked this information [29, 31] or did not found differences for birth weight [25].

There is a need to model lung function development over time in an unsupervised manner, instead of forcing certain structure on the data, and to compare their trajectories applying meta-analytical approaches. Similar trajectory patterns that are identified across various studies will provide critical information on trajectories at higher risk of adverse respiratory development. Having repeated lung function measurements, clinicians then can use such data to early identify and intervene in children/adolescents with risky trajectories.

## Conclusion

The findings of this study add to emerging knowledge of developmental patterns of lung function markers. (1) Men and women and different lung function markers have clearly distinguishable trajectories. (2) We identified lower birth weight, shorter duration of breastfeeding, allergic sensitization at 4 years of age, and, in girls, eczema in infancy and, in boys, asthma at 4 years, as risk factors for lower lung function trajectories, but not parental asthma and maternal smoking during pregnancy. Risk factors vary with the respective lung function marker. Lower lung function trajectories can be explained by both, insufficient growth of airway function until age 18 years and a decline from 18 to 26 years, suggesting that the onset of COPD can arise either from a failure to attain the normal spirometry plateau or from an accelerated decline in lung function [55, 67]. There is a need to integrate findings of multiple studies and of different markers on lung function trajectories.

## Additional file

Additional file 1: **Table S1.** Distribution of forced vital capacity (FVC), forced expiratory flow in one second (FEV1) their ratio (FEV1/FVC), and forced expiratory flow at 25-75% in the pulmonary function trajectories in female and male participants (measurements at 10, 18, and 26 years). **Table S2.** Distribution of participant on trajectories based on at least one and at least two FVC measurements and their cross-tabulation. **Table S3.** Differences in height among the trajectories in girls and boys at age 18 years minus 10 years. (DOCX 21 kb)

## Abbreviations

95% CI: 95% confidence intervals
ALSPAC: Avon Longitudinal Study of Parents and Children
CAMP: Childhood Asthma Management Program
COPD: Chronic obstructive pulmonary disease
FEF_25–75_: Forced expiratory flow 25–75%
FEV_1_: Forced expiratory flow in 1 second
FEV_1_/FVC: Ratio of ratio of FEV_1_ by FVC
FVC: Forced vital capacity
IgE: Immunoglobulin E
IoW: Isle of Wight
MAAS: Manchester Asthma and Allergy Study
OR: Odds ratio
PIAF: Perth Infant Asthma Follow-up
RR: Risk ratio
SPT: Skin prick test
TAHS: Tasmanian Longitudinal Health Study
V’max/FRC: maximal expiratory flow adjusted for functional residual capacity
