# Supplementary material for: Distinctive lung function trajectories from age 10 to 26 years in men and women and associated early life risk factors – a birth cohort study
Source: Respir Res. 2019 May 22;20:98. doi: 10.1186/s12931-019-1068-0 (PMC6532227; doi:10.1186/s12931-019-1068-0)
Supplement: Supplementary file 1 — Table S1. Distribution of forced vital capacity (FVC), forced expiratory flow in one second (FEV1) their ratio (FEV1/FVC), and forced expiratory flow at 25-75% in the pulmonary function trajectories in female and male participants (measurements at 10, 18, and 26 years). Table S2. Distribution of participant on trajectories based on at least one and at least two FVC measurements and their cross-tabulation. Table S3. Differences in height among the trajectories in girls and boys at age 18 years minus 10 years. (DOCX 21 kb) [file 12931_2019_1068_MOESM1_ESM.docx]

**Table S1**: Distribution of forced vital capacity (FVC), forced expiratory flow in one second (FEV1) their ratio (FEV1/FVC), and forced expiratory flow at 25-75% in the pulmonary function trajectories in female and male participants (measurements at 10, 18, and 26 years)

| FVC, liter  Trajectory, men | FVC at age 10 | | | FVC at age 18 | | | FVC at age 26 | | |
| --- | --- | --- | --- | --- | --- | --- | --- | --- | --- |
|  | n | Mean | Standard  deviation | n | Mean | Standard  deviation | n | Mean | Standard  deviation |
| Low | 276 | 2.15 | 0.230 | 213 | 4.83 | 0.432 | 125 | 5.24 | 0.538 |
| High | 212 | 2.61 | 0.283 | 183 | 5.91 | 0.462 | 111 | 6.53 | 0.475 |
| Trajectory, women |  | | | | | | | | |
| Low | 251 | 2.09 | 0.249 | 217 | 3.56 | 0.356 | 159 | 3.84 | 0.356 |
| High | 242 | 2.45 | 0.263 | 226 | 4.34 | 0.351 | 152 | 4.66 | 0.370 |

| FEV1, liter  Trajectory, men | FEV1 at age 10 | | | FEV1 at age 18 | | | FEV1 at age 26 | | |
| --- | --- | --- | --- | --- | --- | --- | --- | --- | --- |
|  | n | Mean | Standard  deviation | n | Mean | Standard  deviation | n | Mean | Standard  deviation |
| Low | 285 | 1.88 | 0.20 | 209 | 4.16 | 0.374 | 124 | 4.08 | 0.492 |
| High | 203 | 2.30 | 0.24 | 187 | 5.12 | 0.403 | 112 | 5.20 | 0.424 |
| Trajectory, women |  | | | | | | | | |
| Low | 239 | 1.80 | 0.211 | 204 | 3.10 | 0.310 | 149 | 3.10 | 0.276 |
| High | 253 | 2.18 | 0.236 | 239 | 3,79 | 0.275 | 162 | 3.72 | 0.301 |

| FEV1/FVC, ratio  Trajectory, men | FEV1/FVC at age 10 | | | FEV1/FVC at age 18 | | | FEV1/FVC at age 26 | | |
| --- | --- | --- | --- | --- | --- | --- | --- | --- | --- |
|  | n | Mean | Standard  deviation | n | Mean | Standard  deviation | n | Mean | Standard  deviation |
| Low | 117 | 0.81 | 0.045 | 103 | 0.77 | 0.049 | 64 | 0.70 | 0.062 |
| High | 371 | 0.90 | 0.045 | 293 | 0.90 | 0.050 | 172 | 0.82 | 0.043 |
| Trajectory, women |  | | | | | | | | |
| Low | 95 | 0.826 | 0.047 | 92 | 0.79 | 0.05 | 71 | 0.74 | 0.049 |
| High | 397 | 0.912 | 0.049 | 351 | 0.90 | 0.049 | 240 | 0.82 | 0.054 |

| Forced expiratory flow at 25-75%, liter  Trajectory, men | FEF25-75 at age 10 | | | FEF25-75 at age 18 | | | FEF25-75 at age 26 | | |
| --- | --- | --- | --- | --- | --- | --- | --- | --- | --- |
|  | n | Mean | Standard  deviation | n | Mean | Standard  deviation | n | Mean | Standard  deviation |
| Low | 233 | 1.98 | 0.352 | 187 | 4.03 | 0.613 | 111 | 3.35 | 0.72 |
| Medium | 266 | 2.68 | 0.405 | 176 | 5.62 | 0.550 | 106 | 5.05 | 0.562 |
| High | 29 | 3.22 | 0.496 | 33 | 7.17 | 0.743 | 19 | 6.53 | 1.005 |
| Trajectory, women |  | | | | | | | | |
| Low | 123 | 1.93 | 0.404 | 116 | 2.91 | 0.485 | 87 | 2.57 | 0.438 |
| Medium | 273 | 2.52 | 0.438 | 235 | 4.03 | 0.438 | 148 | 3.41 | 0.519 |
| High | 97 | 3.07 | 0.428 | 92 | 5.08 | 0.437 | 76 | 4.47 | 0.486 |

**Table S2**: Distribution of participant on trajectories based on at least one and at least two FVC measurements and their cross-tabulation

|  | Male participants  **At least one FVC measurement** | | Female participants  **At least one FVC measurement** | | |
| --- | --- | --- | --- | --- | --- |
| **At least two FVC measurements**  (n=376) | Low FVC-trajectory  (n=329)  column percent | High FVC trajectory  (n=248) column percent | **At least two FVCs** (n=432) | Low FVC trajectory  (n=297)  column percent | High FVC trajectory  (n=283)  column percent |
| Low FVC trajectory (n=214) | 59.57 (n=196) | 0 | Low (n=214) | 75.6 (n=214) | 0 |
| High FVC trajectory (n=218) | 0.6 (n=2) | 71.77 (n=178) | High (n=218) | 1.08 (n=2) | 72.4 (n=218) |
| Not included in this assessment (n=201) | 39.8 (n=131) | 28.2 9 (n=70) | (n=148) | 23.3 (n=66) | 27.8 (n=82) |

Table S3: Differences in height among the trajectories in girls and boys at age 18 years minus 10 years

| Trajectory | | Difference in height (cm) in girls (n=266, n=245)^#^ | p-value in expla­natory models  (Table 2) | p-value of birth weight adju­sting for height | Difference in height (cm) in boys (n=284, n=199) ^#^ | p-value in expla­natory models  (Table 2) | p-value of birth weight adju­sting for height |
| --- | --- | --- | --- | --- | --- | --- | --- |
| FVC trajectories | | 5.52 | 0.04 | 0.004 | 6.01 | 0.02 | 0.95 |
| FEV_1_ trajectories | | 5.00 | <0.0001 | 0.06 | 5.35 | 0.02 | 0.62 |
| FEV_1_/FVC trajectories | | -1.34 | 0.06 | 0.95 | -0.503 | 0.33 | 0.01 |
| FEF_25-75_ trajec­tories | medium vs. high | 3.14 | 0.36 | 0.11 | 4.67 | 0.23 | 0.07 |
|  | low vs. high | 1.36 | 0.008 | 0.04 | 2.48 | 0.002 | 0.01 |

^#^ Number of participants and 10 and 18 years, respectively.
